# Assessing stigma in a predominantly male hemophilia population: a Chinese cross-sectional study

**DOI:** 10.3389/fpsyt.2025.1536880

**Published:** 2025-06-02

**Authors:** Lijuan Ye, Zhuqin Liu, Jianqiong Cao, Yang Wu, Xiao Li, Yanyan Chai

**Affiliations:** Department of Hematology, Nanfang Hospital, Southern Medical University, Guangzhou, China

**Keywords:** hemophilia A, hemophilia B, social stigma, social support, psychology, cross-sectional studies

## Abstract

**Background:**

Hemophilia is a rare hereditary disorder characterized by impaired blood clotting, with prevalence of 2.73 per 100,000 individuals in China. Advancements in medical technology have significantly improved patient survival, however, individuals with hemophilia continue to experience chronic pain, recurrent joint hemorrhages, and functional impairments, particularly in the limbs. These physical challenges often lead to psychological issues such as anxiety and depression, contributing to heightened stigma. This study aimed to assess the current level of stigma among patients with hemophilia and to identify associated influencing factors.

**Methods:**

Convenience sampling was applied to recruit patients from a hemophilia treatment center. As hemophilia predominantly affects males, the sample consisted of 231 males and one female (consistent with epidemiological sex ratios in China). The Social Impact Scale (SIS; 24 items, score range: 24–96, higher scores = greater stigma) and the Social Support Rating Scale (SSRS; 10 items, score range: 12–66, higher scores = greater support) were used to assess stigma and social support, respectively. Multiple linear regression was employed to analyze factors associated with stigma.

**Results:**

The mean total stigma score among hemophilia patients was 63.88 ± 15.27. Multiple linear regression revealed that higher social support (*β* = -0.69, 95% CI [-0.92, -0.47], *P* < 0.001) and being married (*β* = -6.17, 95% CI [-10.06, -2.28], *P* = 0.002) were associated with lower stigma, whereas more frequent bleeding episodes (*β* = 7.79, 95% CI [2.04, 13.54], *P* = 0.008) and lower limb disability (*β* = -6.11, 95% CI [-9.86, -2.36], *P* = 0.002) were associated with higher stigma. Lower monthly income was also linked to increased stigma (*β* = -1.85, 95% CI [-3.58, -0.12], *P* = 0.036). These variables explained 28.9% of the total variation of the total stigma score.

**Conclusions:**

Stigma among patients with hemophilia is at a moderate to severe level. Targeted interventions should be prioritized for unmarried patients, those facing financial hardship, individuals with lower limb disabilities, and those experiencing frequent bleeding episodes. Enhancing psychological health education and social support is essential in mitigating stigma among hemophilia patients.

## 1 Introduction

Hemophilia is a rare hereditary disorder characterized by impaired blood clotting, with a reported prevalence of 2.73 per 100,000 individuals in China (1). Although advances in medical technology have significantly improved survival rates, patients with hemophilia continue to experience chronic pain, recurrent joint hemorrhages, and functional impairments in their limbs. These ongoing physical challenges often lead to psychological issues such as anxiety and depression (2, 3). These physical and mental health challenges contribute to heightened stigma among hemophilia patients (4).

Stigmatization, defined as the negative social perception associated with having a particular disease, is a stress-induced psychological response commonly observed in hemophilia patients (5). A study in Iran found that the physical and social limitations of hemophilia frequently prevent patients from fulfilling social and familial role expectations. These individuals often report fears of rejection, loss of social roles, stigmatization, and discrimination (6), prompting many to conceal their condition (6–8). Stigmatization can severely disrupt interpersonal relationships, lead to social isolation, in extreme cases, increase the risk of suicidal ideation, posing a significant threat to the patient’s well-being (9).

Reinicke et al. highlighted that men with severe hemophilia often face challenges related to masculinity, particularly in their roles as fathers and providers. Limitations in physical activity, including an inability to engage in sports or strenuous tasks, may lead to feelings of inadequacy and frustration, intensifying the experience of stigmatization (8). Similarly, Williams and Chapman emphasize that children with hemophilia face notable peer-related difficulties. The need to conceal their condition often lead to social isolation and challenges in forming close friendships (10).

Despite the substantial impact of stigmatization on individuals with hemophilia, limited research has addressed this issue, both in China and globally. Existing studies are predominantly qualitative in nature. A systematic review by Cassis et al. on the psychosocial aspects of hemophilia highlighted the urgent need for more international research to further explore and quantify the social and psychological aspects of living with the condition (11). This study aims to investigate and analyze the current level and determinants of stigmatization among hemophilia patients. The findings are intended to provide clinical healthcare professionals in developing targeted psychological counseling and interventions tailored to address stigma in this population. We hypothesize that factors such as socioeconomic status, education level, and access to comprehensive healthcare significantly influence the degree of stigma experienced by patients with hemophilia.

## 2 Materials and methods

### 2.1 Study design

This cross-sectional study was conducted at the Hemophilia Registration Center of a tertiary hospital in Guangzhou, China. Patients were recruited using convenience sampling from those under follow-up at the clinic. Of 262 patients under follow-up, 240 volunteered to participate. Informed consent was obtained from all participants prior to completing the questionnaire.

### 2.2 Participants

Inclusion criteria were as follows: (1) Patients diagnosis of hemophilia in accordance with the *Guidelines for the Treatment of Hemophilia in China (2020 Edition)* issued by the Chinese Hemophilia Collaborative Group (12); (2) included both hemophilia A and hemophilia B patients without differentiation; (3) age ≥ 18 years; (4) normal communication ability; (5) capacity to understand the questionnaire; and (6) voluntary participation.

Exclusion criteria included: (1) current or previous diagnosis of mental illness; and (2) presence of other serious underlying medical conditions.

### 2.3 Instruments

Hemophilia Patient General Information Questionnaire: Sociodemographic and disease-related data were collected based on previous literature. Variables included age, sex, height, weight, education level, marital status, occupation, type of medical insurance, monthly family income, type of hemophilia, disease severity, treatment method, presence of joint malformation, and frequency of bleeding episodes.

Social Impact Scale (SIS): The SIS evaluates ill-related stigma and social impact. Originally developed by Fife et al. in 2000 (13), the scale has demonstrated strong reliability (Cronbach’s α = 0.85 - 0.90). It was translated into Chinese by Pan et al. in 2007 (14), and later revised by Guan et al. in 2011 into a 24-item version (15). The revised scale includes social rejection (9 items, People around me discriminated against me because of this disease, I felt like relatives were rejecting me because of the disease, Some people think I’m not as good as I used to be, I feel I’m not as respected as I used to be, The change in my appearance affected how I interacted with others, I think people think I’m to blame for this illness, I’ve had some embarrassing things happen to me because of my illness, I felt that others were worried about getting infected by contact with me, Because of my illness, I felt inequality in my interactions with others), financial insecurity (3 items, The financial difficulties of the disease affected my relationships, The financial difficulties of this illness affected my sense of self, This disease has affected my daily life), internalized stigma (5 items, I felt I needed to keep my illness a secret, I don’t think I’m a healthy person, Because of my illness, sometimes I feel useless, I don’t want people around me to know that I have this disease, I think I’m at least partly to blame for my illness), and social isolation (7 items, I was worried that someone would tell others about my illness without my permission, I felt like people were avoiding me because of the disease, My illness I feel lonelier than ever, I feel like some of my friends are avoiding me because of the disease, More than ever, I need to make sure that people care about me, Compared with before, I feel my ability has decreased, The person next to me seemed nervous and uncomfortable because of my illness). Items are rated on a 4-point Likert scale (1 = strongly disagree to 4 = strongly agree), with higher scores indicating greater perceived stigma (14). The total score ranges from 24 to 96 and is categorized as follows: low stigma (mean score 1.00–1.99; total score 24.0–47.76), moderate stigma (2.00–2.99; 48.0–71.76), and high stigma (3.00–4.00; 72.0–96.0) (16). In this study, the overall Cronbach’s α coefficient was 0.857 (15).

Social Support Rating Scale (SSRS): The scale is developed by Xiao, and was primarily used to assess the level of social support experienced by individuals (17). It includes ten across three distinct dimensions: objective support (items 2-Who you have lived with in the last year, items 6-A source of financial support and help with practical problems that you have received in the past in times of emergency, and items 7- you have been a source of comfort and concern in times of emergency), subjective support (items 1-How many close friends you have that you can get support and help from, items 3-The relationship between you and your neighbors, items 4-Your relationship with your colleagues, and items 5-Support and care received from family members), and support utilization (items 8-The way you talk when you are troubled, items 9-A way to turn to when you are in trouble, and items 10-The frequency of participation in group organization activities). Item scores range form 0–2 or 1-4, depending on the question. Total scores are calculated by summing all items, with higher scores indicating greater social support. Cronbach’s α coefficients for the three dimensions were 0.849, 0.825, and 0.833, respectively.

### 2.4 Data analysis

All statistical analyses were conducted using SPSS version 26.0. Descriptive statistics included frequency, mean, median, standard deviation, distribution normality, and reliability. Correlation and regression analyses were also conducted. A two-sided *P*-value < 0.05 was considered statistically significant.

### 2.5 Ethical considerations

This study was approved by the Ethical Committee of Nanfang Hospital, Southern Medical University (approval number: NFEC-2023-151). All procedures adhere to the ethical principles outlined in the Helsinki Declaration. Written informed consent was obtained from all participants before inclusion in the study.

## 3 Results

### 3.1 Participant characteristics

A total of 240 questionnaires were distributed, and 232 valid responses were collected, resulting in a response rate of 96.7%. Of the respondents, 231 (99.6%) were male and one was female, underscoring the male-dominated nature of hemophilia in our cohort. The mean age was 31.12 ± 11.23 years. Mean height and weight were 169.14 ± 5.34 cm and 62.93 ± 10.89 kg, respectively. Based on BMI, 211 participants (91.0%) were classified as obese. Most participants were unmarried (152, 65.5%). And 78 (33.6%) had completed junior high school. The most common range of per capita monthly family income was 2001–3000 yuan (32.3%). The predominant health insurance type was employee medical insurance (131, 56.5%). Hemophilia A accounted for the majority of case (209, 90.1%), and 170 patients (73.3%) had severe hemophilia. Inhibitors presence was reported in 201 patients (88.6%). The most common treatment approach was on-demand plus prophylaxis (91, 39.2%). Lower limb dysfunction and arthritis were reported in 136 (58.6%) and 172 (74.1%) patients. While most patients did not require assistive devices, 188 (81.0%) reported using them, and 172 (74.1%) had experience a fall. 210 patients (90.5%) had reported 50 or fewer episodes of bleeding in the past 12 months. The mean pain score was 4.24 ± 2.45, and 102 (44.0%) reported moderate pain.

### 3.2 The current status of stigma and social support

The total and item-level scores for stigma in hemophilia patients, and levels of social support are detailed in **Table 1**. Single-factor analysis revealed that stigma scores varied significantly (*P* < 0.05) by several factors, including marital status, average monthly income per family member, payment method for medical expenses, treatment regimen, presence of disabled joints in the lower limbs, history of arthritis, history of falls, and the number of bleeding episodes in the past 12 months, as detailed in **Table 2**. Variables such as age, gender, height, weight, education level, employment status, type of health insurance, type of hemophilia, and severity did not have a significant impact stigma scores (*P* > 0.05). The analysis of illness stigma across four dimensions revealed significant differences in social exclusion, which varied with marital status, per capita monthly household income, medical payment method, lower limb disability, use of assistive devices, history of falls, number of bleeds in the past year, and pain level. Intrinsic stigma showed significant variation with marital status, lower extremity disability, lower extremity arthritis, and number of bleeds in the past year. Economic discrimination differed significantly by marital status, per capita monthly household income, treatment program, disabled joints of the lower extremities, arthritis of the lower extremities, and number of bleeds in the past year. Social segregation varied significantly with marital status, per capita monthly household income, treatment programs, arthritis of the lower extremities, use of assistive devices, history of falls, number of bleeds in the past year, and pain level.

**Table 1:** Stigma levels and social support status in patients with hemophilia (Mean ± SD, N=232).

Item	Total/dimension scores	Item score mean
SIS Dimensions		
Social influence	63.88 ± 15.27	2.66+/-0.64
Social exclusion (9 item)	24.58 ± 6.07	2.73+/-0.67
Intrinsic stigma (5 item)	15.36 ± 4.33	2.56+/-0.72
Economic discrimination (3 item)	7.81 ± 2.31	2.60+/-0.77
Social isolation (7 item)	16.14 ± 4.07	2.69+/-0.68
SSRS Dimensions		
Social support assessment	34.28 ± 8.04	2.45+/-0.57
Objective support (items 2, 6, 7)	7.87 ± 2.27	2.62+/-0.76
Subjective support (items 1, 3, 4, 5)	20.09 ± 5.78	2.51+/-0.72
Support availability (items 8, 9, 10)	6.33 ± 2.25	2.11+/-0.75

SIS (Stigma Impact Scale) Dimensions: Social influence, Social exclusion, Intrinsic stigma, Economic discrimination, Social isolation.

SSRS (Social Support Rating Scale) Dimensions: Social support assessment, Objective support, Subjective support, Support availability.

**Table 2:** Comparison of stigma scores among hemophilia patients with different demographic and disease characteristics (N=232).

Item	Cases (n%)	Stigma Score			Social exclusion			Intrinsic stigma			Economic discrimination			Social segregation		
		(X ± s)	T/F Value	P Value	(X ± s)	T/F Value	P Value	(X ± s)	T/F Value	P Value	(X ± s)	T/F Value	P Value	(X ± s)	T/F Value	P Value
Age (years)			0.737	0.480		1.371	0.256		0.726	0.485		0.020	0.980		0.482	0.618
18-44	202 (87.1)	63.37 ± 15.79			24.42 ± 6.16			15.36 ± 4.40			7.81 ± 2.41			16.08 ± 4.24		
45-59	25 (10.8)	66.68 ± 11.26			26.32 ± 5.41			15.76 ± 3.97			7.80 ± 1.66			16.80 ± 2.84		
≥60	5 (2.1)	58.60 ± 9.50			22.60 ± 4.83			13.20 ± 2.77			7.60 ± 0.55			15.20 ± 1.64		
BMI			1.499	0.226		0.590	0.555		1.848	0.16		2.760	0.065		2.110	0.124
<18.5	4 (1.7)	70.75 ± 11.30			26.75 ± 5.19			17.00 ± 3.16			9.00 ± 0.82			18.00 ± 2.45		
18.5-24.9	17 (7.3)	69.00 ± 14.23			25.63 ± 5.19			17.00 ± 4.38			8.84 ± 1.77			17.74 ± 2.96		
25-29.9	211 (91.0)	63.34 ± 15.36			24.45 ± 5.19			15.18 ± 4.32			7.69 ± 2.34			15.96 ± 4.15		
Educational level			1.525	0.209		1.410	0.241		0.260	0.854		2.321	0.076		2.524	0.058
Primary school and below	50 (21.6)	62.92 ± 17.82			24.02 ± 6.81			15.14 ± 4.89			7.86 ± 2.68			15.90 ± 4.71		
Junior school	78 (33.6)	63.95 ± 13.37			24.55 ± 5.81			15.44 ± 3.72			7.90 ± 2.04			16.06 ± 3.57		
High school	44 (19.0)	67.91 ± 12.97			26.18 ± 5.24			15.80 ± 4.26			8.39 ± 2.10			17.55 ± 3.43		
undergraduate and above	60 (25.8)	61.65 ± 16.59			23.92 ± 6.25			15.12 ± 4.70			7.22 ± 2.37			15.40 ± 4.38		
Marital Status			3.118	0.002		3.165	0.002		2.528	0.012		2.081	0.039		3.073	0.002
Marital Status	80 (34.5)	68.11 ± 13.36			26.29 ± 5.62			16.34 ± 3.92			8.24 ± 2.09			17.25 ± 3.49		
Unmarried	152 (65.5)	61.66 ± 15.77			23.68 ± 6.12			14.84 ± 4.46			7.58 ± 2.39			15.55 ± 4.24		
Monthly Household Income per Capita (CNY/month)			4.343	0.005		4.225	0.006		1.734	0.161		3.728	0.012		5.091	0.002
<2001	64 (27.6)	68.81 ± 15.82			26.52 ± 6.23			16.27 ± 4.71			8.52 ± 2.32			17.52 ± 4.25		
2001~3000	75 (32.3)	63.09 ± 15.06			24.36 ± 6.09			15.12 ± 4.29			7.72 ± 2.42			15.89 ± 4.05		
3001~5000	65 (28.0)	62.80 ± 13.12			24.05 ± 5.18			15.25 ± 3.89			7.58 ± 1.95			15.92 ± 3.34		
>5000	28 (12.1)	57.25 ± 16.51			22.00 ± 6.55			14.18 ± 4.36			6.93 ± 2.39			14.14 ± 4.41		
Types of disease			0.278	0.782		0.014	0.989		0.619	0.536		0.812	0.417		-0.098	0.922
Hemophilia A	209 (90.1)	63.98 ± 15.51			24.58 ± 6.21			15.42 ± 4.38			7.85 ± 2.34			16.13 ± 4.11		
Hemophilia B	23 (9.9)	63.04 ± 13.15			24.57 ± 6.21			14.83 ± 3.92			7.43 ± 1.97			16.22 ± 3.78		
Severity of hemophilia			1.509	0.223		2.682	0.071		1.158	0.316		0.414	0.662		0.762	0.468
Mild hemophilia	11(4.7)	62.27 ± 12.82			23.09 ± 4.74			15.82 ± 2.79			7.36 ± 2.58			16.00 ± 4.05		
Moderate hemophilia	51(22.0)	60.78 ± 14.14			23.06 ± 5.34			14.55 ± 4.26			7.65 ± 2.11			15.53 ± 3.78		
Severe hemophilia	170 (73.3)	64.92 ± 15.67			25.14 ± 6.28			15.57 ± 4.42			7.88 ± 2.35			16.33 ± 4.16		
Profile of Inhibitors			0.572	0.568		0.646	0.519		0.369	0.712		0.544	0.587		0.480	0.631
Yes	26 (11.2)	65.50 ± 14.00			25.31 ± 5.30			15.65 ± 4.20			8.04 ± 2.13			16.50 ± 3.62		
No	206 (88.8)	63.68 ± 15.44			24.49 ± 5.30			15.32 ± 4.36			7.78 ± 2.33			16.09 ± 4.13		
Use of assistive products			1.787	0.075		2.609	0.010		-0.144	0.885		0.982	0.327		2.450	0.015
Yes	44 (19.0)	67.57 ± 15.35			24.09 ± 6.11			15.27 ± 4.91			8.11 ± 2.66			17.48 ± 3.92		
No	188 (81.0)	63.02 ± 15.16			21.20 ± 6.10			15.38 ± 4.20			7.73 ± 2.22			15.82 ± 4.05		
Medical payment method			2.722	0.045		4.886	0.003		0.759	0.518		0.771	0.512		2.586	0.054
Urban-Rural Resident Insurance	90 (38.8)	60.78 ± 16.44			23.02 ± 6.47			14.89 ± 4.42			7.61 ± 2.37			15.26 ± 4.39		
Employee Insurance	131 (56.5)	66.36 ± 14.37			25.86 ± 5.58			15.73 ± 4.40			7.99 ± 2.29			16.78 ± 3.78		
Public Medical Care	3 (1.3)	58.33 ± 5.51			20.00 ± 6.93			15.00 ± 1.73			7.33 ± 0.58			16.00 ± 0.00		
Self-funded	8 (3.4)	60.38 ± 12.19			23.00 ± 4.31			14.63 ± 2.33			7.13 ± 2.17			15.63 ± 4.31		
Treatment Plan			4.641	0.011		3.680	0.027		2.388	0.094		3.116	0.046		6.974	0.001
On-demand Treatment	63 (27.2)	65.17 ± 14.25			24.98 ± 5.63			15.43 ± 3.99			8.13 ± 2.17			16.63 ± 3.84		
Preventive Treatment	78 (33.6)	59.73 ± 17.01			23.12 ± 6.74			14.55 ± 4.69			7.28 ± 2.46			14.78 ± 4.48		
Combination of On-demand and Preventive	91 (39.2)	66.55 ± 13.07			25.56 ± 5.56			16.00 ± 4.17			8.03 ± 2.20			16.96 ± 3.57		
Disability in Lower Limbs			5.224	<0.001		5.979	<0.001		2.854	0.005		3.513	0.001		5.657	<0.001
Yes	136 (58.6)	68.05 ± 13.87			26.45 ± 5.46			16.03 ± 4.32			8.24 ± 2.27			17.33 ± 3.60		
No	96 (41.4)	57.98 ± 15.27			21.94 ± 5.93			14.41 ± 4.20			7.19 ± 2.23			14.45 ± 4.12		
Arthritis			4.487	<0.001		5.297	<0.001		2.144	0.033		2.724	0.007		5.174	<0.001
Yes	172 (74.1)	66.44 ± 14.16			25.76 ± 5.62			15.72 ± 4.30			8.05 ± 2.19			16.91 ± 3.71		
No	60 (25.9)	56.57 ± 16.05			21.20 ± 6.10			14.33 ± 4.28			7.12 ± 2.51			13.92 ± 4.28		
History of Falls			2.259	0.025		2.368	0.019		1.754	0.081		1.588	0.114		2.163	0.032
Yes	172 (74.1)	65.21 ± 15.05			25.13 ± 5.97			15.65 ± 4.33			7.95 ± 2.27			16.48 ± 3.89		
No	60 (25.9)	60.08 ± 15.37			23.00 ± 6.12			14.52 ± 4.27			7.40 ± 2.39			15.17 ± 4.45		
Bleeding Episodes in the Past 12 Months			-3.041	0.003		-2.547	0.012		-2.518	0.012		-2.380	0.018		-3.551	<0.001
Below 50 times	210 (90.5)	62.91 ± 15.02			24.26 ± 5.98			15.13 ± 4.26			7.69 ± 2.28			15.84 ± 4.00		
Below 50 times	22 (9.5)	73.14 ± 14.78			27.68 ± 6.18			17.55 ± 4.48			8.91 ± 2.29			19.00 ± 3.74		

### 3.3 Correlation of stigma and social support rating scale in hemophilia patients

Pearson correlation analysis demonstrated statistically significant inverse relationships between stigma and social support dimensions. The strength of these associations varied: A moderate negative correlation was observed for support utilization (*r* = -0.428) and total social support score (*r* = -0.409), indicating that patients with better support systems reported substantially lower stigma levels. Subjective support showed a weaker but still meaningful negative association (*r* = -0.327).Objective support exhibited the weakest, albeit significant, correlation (*r* = -0.191).

### 3.4 Factors influencing the stigma of hemophilia patients

Multiple linear stepwise regression was conducted using stigma score as the dependent variable. Independent variables included marital status (married = 1; unmarried = 2), family per capita monthly income (<2000 yuan/month [≈280 USD] = 1; 2001–3000 yuan/month [≈280–420 USD] = 2; 3001–5000 yuan/month [≈420–700 USD] = 3; >5000 yuan/month [>700 USD] = 4; based on 2023 average exchange rate: 1 CNY = 0.14 USD, State Administration of Foreign Exchange, 2023), medical approach (urban and rural residents’ insurance = 1; employee’s medical insurance = 2; public funding = 3; self-funded = 4), treatment plan (treatment on demand = 1; prophylactic treatment = 2; combination of treatment on demand and prophylaxis = 3), presence of a disabled joint in the lower limb (yes = 1; no = 2), presence of arthritis (yes = 1; no = 2), history of falls (yes = 1; no = 2), the number of bleeding episodes in the past 12 months (less than 50 times = 1; more than 50 times = 2), pain sensation (a continuous random variable), and social support assessment (a continuous random variable).

The final model (adjusted *R²* = 0.289, *F* = 19.797, *p* < 0.05), identified the following as significant predictors of stigma: marital status (95% CI [-10.06, -2.28], *P =* 0.002), monthly family income (95% CI [-3.58, -0.12], *P* = 0.036), presence of a disabled joint in the lower limb (95% CI [-9.86, -2.36], *P* = 0.002), and social support assessment (95% CI [-0.92, -0.47], *P* < 0.001), and number of bleeding episodes in the past 12 months (95% CI [2.04, 13.54], *P* = 0.008) (see **Table 3**).

**Table 3:** Multiple linear stepwise regression analysis of hemophilia patients.

Independent variables	Stigma Score *(R²* = 0.289)				social exclusion (*R^2^ =* 0.291)				Intrinsic stigma (*R^2^ =* 0.147)				Economic discrimination (*R^2^ =* 0.191)				Social segregation (R^2^ =0.295)			
	B Value	β Value	95%CI (B)	p Value	B Value	β Value	95%CI (B)	p Value	B Value	β Value	95%CI (B)	p Value	B Value	β Value	95%CI(B)	p Value	B Value	β Value	95%CI (B)	p Value
(Constant)	101.70		89.20;114.21	<0.001	39.956		32.166;47.746	<0.001	22.875		19.013;26.738	<0.001	12.763		10.637;14.890	<0.001	25.307		20.450;30.165	<0.001
Marital Status	-6.16	-0.19	-10.06;-2.28	0.002	-2.102	-0.165	-3.689;-0.514	0.010	-1.772	-0.195	-2.991;-0.552	0.005	-0.741	-0.153	-1.381; -0.102	0.023	-1.425	-1.251	-2.480;-0.369	0.008
Monthly Household Income per Capita	-1.85	-0.12	-3.58;-0.12	0.036									-0.292	-0.125	-0.574; -0.010	0.042	-0.527	2.488	-0.993;-0.061	0.027
Disability in Lower Limbs	-5.80	-0.19	-9.86;	0.002	-2.034	-0.165	-3.853;-0.215	0.029									-1.251	-0.161	-2.436;-0.061	0.039
Bleeding Episodes in the Past 12 Months	7.79	0.15	2.04;-2.36	0.008					-1.772	0.127	0.082;3.654	0.040					2.488	-1.251	0.932;4.044	0.002
Social Support Assessment	-0.69	-0.37	-0.92;-0.47	<0.001	-0.248	-0.328	-0.338;-0.158	<0.001	-0.172	-0.319	-0.240;-0.104	<0.001	-0.098	-0.342	-0.134;-0.063	<0.001	-0.161	2.488	-0.221;-0.101	<0.001

The model of Social Exclusion(adjusted *R²* = 0.291, *F* = 8.894, *p* < 0.05) identified the following as significant predictors of social exclusion: marital status (95% CI [-3.689, -0.514], *P* = 0.010), presence of a disabled joint in the lower limb (95% CI [-3.853, -0.215], *P* = 0.029), and social support assessment (95% CI [-0.338, -0.158], *P* < 0.001).The model of Internalized stigma(adjusted *R²* = 0.147, *F* = 8.955, *p* < 0.05) identified the following as significant predictors of internalized stigma: marital status (95% CI [-2.991, -0.552], *P* = 0.005), number of bleeding episodes in the past 12 months (95% CI [0.082, 3.654], *P* = 0.040), and social support assessment (95% CI [-0.240, -0.104], *P* < 0.001).The model of Economic Discrimination(adjusted *R²* = 0.191, *F* = 8.794, *p* < 0.05) identified the following as significant predictors of economic discrimination: marital status (95% CI [-1.381, -0.102], P = 0.023), monthly family income (95% CI [-0.574, -0.010], *P* = 0.042), and social support assessment (95% CI [-0.134, -0.063], *P* < 0.001).The model of Social Isolation(adjusted *R²* = 0.295, *F* = 10.665, *p* < 0.05) identified the following as significant predictors of social isolation: marital status (95% CI [-2.480, -0.369], *P* = 0.008), monthly family income (95% CI [-0.993, -0.061], *P* = 0.027), presence of a disabled joint in the lower limb (95% CI [-2.436, -0.066], P = 0.039), number of bleeding episodes in the past 12 months (95% CI [0.932, 4.044], *P* = 0.002), and social support assessment (95% CI [-0.221, -0.101], *P* < 0.001) (see **Table 3**).

## 4 Discussion

This study indicated that patients with hemophilia experience moderate to severe levels of stigma, higher than levels reported by Bulgin et al. in patients with genetic hematological diseases such as sickle cell disease (18). This may be attributed to the additional burdens of joint bleeding, disability, and dependency on family members, which exacerbate feelings of stigma.

Among stigma dimensions, the highest to lowest were: social exclusion, social isolation, internalized stigma, and economic discrimination. These findings are consistent with those reported by Li et al. (19). The hierarchy of these dimensions may be associated with the physical activity limitations and disabilities caused by hemophilia, which often lead patients to face societal barriers and challenges related to unemployment. Such experiences can instill a fear of losing their social roles, thereby contributing to internal stigma (6). For instance, a study by Barta et al. (20) found that individuals with chronic illnesses who perceive themselves as unable to fulfill societal roles are more likely to experience internalized stigma, which aligns with our findings.

Additionally, social isolation may result from the protective family behaviors, which can diminish the patients’ self-care abilities and reduce their participation in social activities (21). This phenomenon is aligned with Martire et al. (22), who noted that overprotective family behaviors, while well-intentioned, can inadvertently reinforce feelings of dependency and isolation in patients with chronic illnesses.

Although economic discrimination was ranked lowest in our study, it remains a significant concern. The financial strain associated with hemophilia treatment and the potential loss of employment due to physical limitations can exacerbate feelings of stigma, particularly in low-income populations (23). Consequently, these results underscore the clinical need for routine stigma screening coupled with targeted interventions should include: Structured family education (24), Peer-led support groups to counteract social isolation and Clinician stigma awareness trainings (17).

Marital status significantly affected stigma level, and affected all dimensions of stigma. Specifically, married patients report lower levels of stigma compared to their unmarried counterparts, consistent with findings by Wu et al. (25). This difference may be due to the emotional and practical support that married patients receive from their spouses, which can mitigate internalized stigma. Additionally, spousal support may also reduce social isolation and loneliness, thereby further reducing overall feelings of stigma (26).

Conversely, Tang et al. (27) argued that unmarried patients often experience more intense stigma due to the absence of partner support, fewer confidants, and limited social support. Accordingly, healthcare professionals should evaluate stigma levels of unmarried hemophilia patients, encouraging their engagement in social activities, helping them establish meaningful social connections, and facilitating access to additional social support can help compensate for the lack of spousal support and reduce their feelings of stigma.

From an economic perspective, the household per capita monthly income was negatively correlates with stigma, in line with the findings of Gong et al. (28). This relationship may be reflect the chronic nature of hemophilia, which requires lifelong treatment with clotting factors. Long-term treatment needs and potential employment disruption can increase the family’s economic burdens and foster feelings of guilt and stigma in patients (29).

Research indicates that lower income may also affect a patient’s social status, making them more vulnerable to societal discrimination (30). Although hemophilia treatment is covered under China’s rare disease medical reimbursement system, the cost of managing complications still impose financial strain on families. Inadequate treatment may lead to hemophilic arthropathy and disability.

However, its association with internalized stigma was nonsignificant, indicating that economic constraints may not directly increase self-stigma but instead operate indirectly by restricting social participation or employment opportunities (29, 31). Income significantly predicted social exclusion in univariate analysis, but this effect diminished in multivariate models, likely due to collinearity with social support, which exerted a stronger protective effect.

This study also indicated that hemophilia patients with disabled joints in their lower limbs experience higher levels of stigma, consistent with the findings of Liu et al. (32). This may be associated with the functional limitations, abnormal gait, and perceived inferiority. Dong et al. (33) found that the severity of motor dysfunction correlates with more pronounced negative emotions, including deeper experiences of stigma. Additionally, studies have shown that joint deformities or disabilities can increase the psychological burden on hemophilia patients, adversely affecting their social interactions and making them more susceptible to feelings of social isolation (34). Contrary to Liu et al. (32), lower limb disability showed no correlation with internalized stigma. This discrepancy could stem from cultural influences; in collectivist societies, family support may mitigate self-stigma despite physical limitations, while external stigmatization continues (35).

To address these challenges, clinical healthcare providers should offer appropriate exercise rehabilitation guidance to hemophilia patients and focus on building their confidence, thereby helping to alleviate their feelings of stigma.

Furthermore, the number of bleeding episodes in the past 12 months positively influences the level of stigma among patients with hemophilia. A likely explanation for this relationship is that more frequent bleeding events reflect more severe illness, increasing the psychological burden and leading to negative emotions (35). These findings highlight the importance of effective disease management in reducing both the physical and psychological burdens associated with hemophilia. Harris et al. (36) found that proactive management of bleeding episodes through regular prophylaxis significantly reduces both the frequency of bleeding and the psychological distress associated with it, offering potential benefit for hemophilia patients. However, bleeding frequency did not significantly predict economic discrimination and social exclusion, possibly because financial stress in hemophiliacs was more closely associated with long-term treatment costs than acute episodes (36). In addition, social exclusion is more likely to result from a combination of economic hardship, physical disability and psychological burden caused by the disease.

Social support was found to be a strong protective factor against stigma, consistent with the findings of Zheng et al. (37). This relationship may stem from the fact that increased social support helps hemophilia patients experience less psychological stress. Social support includes both emotional and material assistance from the patients’ environment. Emotional support can promote feelings of respect and reduce experiences of social isolation and discrimination, while material support can alleviate the financial burden on patients’ families, thereby diminishing feelings of guilt (19).

Support from family and friends may also enhance feelings of belonging and respect, and fulfil social needs, minimizing social isolation and exclusion due to role changes, and reducing their internal stigma (31). Therefore, healthcare professionals should prioritize frequent communication with patients to foster positive relationships. They should also provide comprehensive information about hemophilia and guide the families and friends of patients in offering adequate care, ultimately helping to reduce stigma among patients.

### 4.1 Limitations

This study has several limitations. First and foremost, our sample consisted of 231 males (99.6%) and only one female (0.4%), reflecting the X-linked recessive inheritance pattern of hemophilia. While this gender distribution is biologically expected, it precludes meaningful analysis of stigma experiences in female patients or carriers. Future studies should make targeted efforts to recruit female participants to understand potential gender differences in stigma perception. Second, the sample was drawn from a single province, which may limit generalizability of the findings to a wider population. Future research should utilize multicenter designs to capture broader demographic and clinical variations. Third, this study did not account for mental health status or disease duration, which may have influenced the regression model and led to an underestimation of stigma levels. Future studies should incorporate these variables to provide a more comprehensive assessment. Additionally, the cross-sectional design limits causal inferences; longitudinal or interventional studies are recommended.

## 5 Conclusion

The study reveals that hemophiliacs experience moderate to severe stigma, influenced primarily by marital status, household income, lower limb joint disability, and annual bleeding frequency. However, these factors differentially affect stigma dimensions: marital status and social support impact all stigma domains, while income predominantly influences economic hardship and social isolation. Physical disability primarily exacerbates social exclusion and isolation but minimally affects intrinsic self-stigma. Bleeding frequency correlates with intrinsic stigma and social isolation. Hence, healthcare professionals should implement targeted interventions to address these vulnerabilities. Enhancing social support networks, especially for unmarried individuals, can mitigate feelings of isolation and stigma. Providing financial aid and promoting stigma awareness initiatives can benefit low-income families. Patients experiencing frequent bleeding or physical disabilities may require comprehensive rehabilitation and psychosocial assistance.

## Data Availability

The raw data supporting the conclusions of this article will be made available by the authors, without undue reservation.
